# Efficient Extraction of Coronary Artery Vessels from Computed Tomography Angiography Images Using ResUnet and Vesselness

**DOI:** 10.3390/bioengineering11080759

**Published:** 2024-07-26

**Authors:** Omar Ibrahim Alirr, Hamada R. H. Al-Absi, Abduladhim Ashtaiwi, Tarek Khalifa

**Affiliations:** 1 College of Engineering and Technology, American University of the Middle East, Egaila 54200, Kuwaittarek.khalifa@aum.edu.kw (T.K.); 2 College of Science and Engineering, Hamad Bin Khalifa University, Doha 34110, Qatar

**Keywords:** deep learning, coronary artery segmentation, U-net, CTA images, cardiovascular disease

## Abstract

Accurate and efficient segmentation of coronary arteries from CTA images is crucial for diagnosing and treating cardiovascular diseases. This study proposes a structured approach that combines vesselness enhancement, heart region of interest (ROI) extraction, and the ResUNet deep learning method to accurately and efficiently extract coronary artery vessels. Vesselness enhancement and heart ROI extraction significantly improve the accuracy and efficiency of the segmentation process, while ResUNet enables the model to capture both local and global features. The proposed method outperformed other state-of-the-art methods, achieving a Dice similarity coefficient (DSC) of 0.867, a Recall of 0.881, and a Precision of 0.892. The exceptional results for segmenting coronary arteries from CTA images demonstrate the potential of this method to significantly contribute to accurate diagnosis and effective treatment of cardiovascular diseases.

## 1. Introduction

Coronary artery disease (CAD) is a prevalent and serious condition caused by the gradual accumulation of plaque in the arteries, leading to a restricted or blocked blood supply to the heart. This can cause symptoms like chest pain (angina) and greatly elevate the risk of a heart attack, which is a major contributor to global mortality. Managing CAD requires a comprehensive strategy, encompassing lifestyle modifications, risk factor control, pharmacological treatment, and occasionally interventional procedures or surgery. Precise diagnosis and surveillance of CAD are essential for prompt treatment and enhanced patient prognosis [1,2,3,4].

One of the key diagnostic tools for CAD is Computed Tomography Angiography (CTA), which generates high-resolution images of the coronary arteries. Manually segmenting these arteries from CTA images is crucial in clinical settings as it allows healthcare providers to assess the extent and severity of coronary artery disease. However, this manual segmentation process faces several challenges that impact its accuracy, efficiency, and consistency. Manually segmenting coronary arteries from CTA images is a time-consuming and labor-intensive task, requiring highly skilled medical professionals to manually delineate the artery boundaries. This process can take hours per case, significantly straining healthcare resources and delaying patient care delivery. Additionally, different medical professionals may have varying interpretations and segmentation criteria, leading to inconsistent results. Even the same professional may produce dissimilar segmentation outcomes when performing the task repeatedly, compromising the reproducibility and accuracy of the process [5,6].

Overcoming these challenges is crucial for enhancing the quality of cardiovascular imaging and patient care. Automated, reliable, and accurate coronary artery segmentation methods can greatly improve the efficiency and consistency of this crucial diagnostic task, allowing healthcare professionals to allocate more time to patient care and decision making [7,8,9]. Recent progress in deep learning techniques has shown immense potential in transforming the field of medical image analysis, including the segmentation of coronary arteries from CTA images. Deep learning models, particularly the widely used U-Net architecture, have exhibited exceptional performance in accurately segmenting coronary arteries, surpassing conventional segmentation methods [10,11,12,13,14].

Beyond deep learning-based methods, some studies have explored hybrid techniques that integrate deep learning with conventional image processing approaches. For instance, Mihalef et al. developed a method that combines a level set function with deep learning for precise coronary artery segmentation, harnessing the advantages of both techniques to tackle the challenges in CTA image analysis. Additionally, Gao et al. proposed an automated approach to segment the three-dimensional coronary tree from Computed Tomography Angiography (CTA) images [15]. The method combines a learning-based approach with graph-cut optimization to segment the vessels. The results demonstrated the method’s ability to accurately segment the major coronary arteries and their branches, enabling a comprehensive analysis of the coronary tree. Wolterink et al. developed a graph neural network approach for automated extraction and labeling of the coronary artery tree from CTA images. The method employs a deep learning model to predict the presence and labels of coronary arteries at each voxel. The predicted labels are then used to construct a graph representation of the coronary tree. The study highlighted the effectiveness of graph neural networks in accurately extracting and labeling the coronary arteries [16].

Overall, deep learning-based methods, particularly those incorporating attention mechanisms and hybrid approaches, have demonstrated promising results in improving the accuracy, efficiency, and consistency of this critical task. The automatic segmentation of the complete coronary tree and the use of graph neural networks for the extraction and labeling of the coronary arteries further enhance the comprehensive analysis of the coronary vasculature. However, the field of coronary artery segmentation from CTA images still faces several challenges, including the presence of noise and artifacts in CTA images, which can negatively impact the accuracy of segmentation algorithms. Additionally, the lack of publicly available datasets for this task has hindered the progress and widespread adoption of these advanced techniques [17].

This study aims to tackle these pressing challenges by proposing an advanced deep learning framework that incorporates vesselness features for accurate and efficient segmentation of coronary arteries from CTA images. By harnessing the power of deep learning and incorporating innovative architectural elements, we seek to push the boundaries of what is currently achievable in this critical field of cardiovascular imaging and patient care. The successful implementation of this framework has the potential to significantly transform the way healthcare professionals approach the diagnosis, treatment, and monitoring of coronary artery disease. Accurate and efficient coronary artery segmentation can enable earlier detection of atherosclerosis, more targeted interventions, and improved patient outcomes, ultimately contributing to the overall well-being of individuals affected by this prevalent cardiovascular condition.

## 2. Materials and Methods

**Figure 1:**
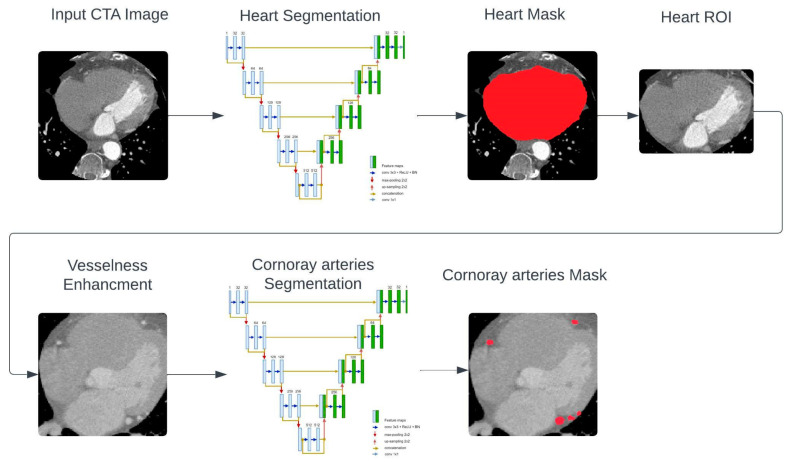
Mainstages of the proposed method.

The proposed segmentation system focuses on the heart region of interest (ROI) within the target CTA scan. This work employed a structured pipeline consisting of several key steps forming the proposed framework, as shown in Figure 1. These steps leverage advanced techniques, including vesselness, heart ROI extraction, and the ResUNet deep learning method, to achieve accurate and efficient extraction of coronary artery vessels, crucial for the diagnosis and treatment of cardiovascular diseases. Vesselness filtering serves as a pivotal technique for enhancing the visibility of coronary arteries in CTA images. By applying vessel enhancement filters using the Frangi filter, vessel-like structures can be highlighted while background noise is suppressed. This step significantly improves the detectability of coronary arteries within the image data.

Subsequently, extracting the region of interest (ROI) corresponding to the heart becomes essential for directing the segmentation process towards the coronary arteries. Heart ROI extraction involves the identification of the cardiac region within the CTA images, achieved through a deep learning-based approach. By isolating the heart region, segmentation algorithms can focus exclusively on extracting coronary arteries with heightened precision.

Finally, the ResUNet model, a hybrid architecture combining Residual Networks (ResNet) and U-Net, emerges as a powerful tool for coronary artery segmentation from CTA images. This sophisticated deep learning model enhances both the accuracy and efficiency of segmentation tasks, particularly in handling complex anatomical structures. By leveraging the strengths of ResNet and U-Net architectures, ResUNet proves instrumental in achieving robust and reliable segmentation results in the challenging domain of cardiovascular imaging.

### 2.1. Clinical Datasets

This work utilized datasets from two different public sources: the CTA-ASOCA and MM-WHS datasets. These are two prominent datasets used in the fields of coronary artery segmentation and multi-modality whole heart segmentation, respectively. The CTA-ASOCA dataset is a publicly available dataset provided by the ASOCA challenge for coronary artery segmentation. It consists of 40 CTA (Coronary Computed Tomography Angiography) images, including 20 healthy cases and 20 patients with confirmed coronary artery disease. In contrast, the MM-WHS 2017 dataset is a dataset for multi-modality whole-heart segmentation, providing 20 labeled and 40 unlabeled CT volumes, as well as 20 labeled and 40 unlabeled MR volumes, for a total of 120 multi-modality cardiac images acquired in a real clinical environment. The MM-WHS 2017 dataset is used for the Multi-Modality Whole Heart Segmentation Challenge, which aims to create an open and fair competition for various research groups to test and validate their methods, particularly for the task of multi-modality whole heart segmentation.

The CTA-ASOCA dataset is significant because it provides a benchmark for coronary artery segmentation, which is essential for diagnosing and quantifying coronary artery disease. The dataset includes annotations produced by three expert annotators for the training set, enabling a comparison and ensuring the reproducibility of results. Additionally, the dataset is large-scale, with 40 CTA images, and is publicly available, which allows for comparisons to be made and ensures the reproducibility of results.

The MM-WHS dataset is significant because it provides a platform for researchers to develop and evaluate their methods for multi-modality whole heart segmentation. The dataset includes 120 multi-modality cardiac images acquired in a real clinical environment, allowing for the development of computer-aided diagnosis of coronary artery diseases that can handle real-world data. The dataset was also used for the Multi-Modality Whole Heart Segmentation Challenge 2017, facilitating comparisons and ensuring the reproducibility of results.

### 2.2. Data Pre-Processing

Preprocessing is a crucial step in the analysis of Coronary Computed Tomography Angiography (CTA) images to enhance image quality and standardize data for accurate deep learning-based segmentation of coronary arteries. This process involves several steps, including image resampling, noise reduction, contrast enhancement, and normalization.

The image resampling step aims to ensure that the images are uniformly spaced and aligned, which improves the accuracy of the segmentation process. We applied the resampling technique on CTA input images to achieve a 1 mm spacing, which was set as the standard resolution for all CTA images. This step involves interpolating the image data to create new voxels with a uniform spacing of 1 mm, facilitating the segmentation process and ensuring that the segmented arteries are aligned and uniformly spaced.

Furthermore, some CTA images may contain noise arising from various sources, such as image acquisition, patient motion, and image reconstruction. The noise reduction step aims to remove or reduce the effects of noise on the image data, which improves the accuracy of the segmentation process. For this purpose, we used anisotropic diffusion filtering as the noise reduction technique. Anisotropic diffusion filtering is a non-linear filtering technique that preserves the edges and details in the image while reducing noise. This technique is effective for removing noise while preserving the anatomical details of the coronary arteries.

Diffusion filtering utilizes a structure tensor to characterize image structure, employing features or local coherence to guide the diffusion process [18]. Mendrik et al. introduced two types of structure tensors: coherence enhancing diffusion (CED) and edge enhancing diffusion filters (EED). CED is particularly effective for enhancing tube-like structures compared to EED. Given its focus on tube-like structures, CED operates as one-dimensional diffusion, potentially diffusing in a single direction or not at all. In the context of the structure tensor, if the eigenvalues (µ1 > µ2 > µ3) align with the eigenvectors V1, V2, V3, CED conducts diffusion along the V3 direction. The ratio between the second and third eigenvalues determines whether diffusion occurs. This ratio is significant for vessel-like structures and minimal for blob or plate-like structures [19].

Normalization is another step in the preprocessing stage of using CTA images for coronary artery segmentation. CTA scans could come with different ranges of intensities; hence the normalization technique aims to standardize the image data by adjusting the intensity values to a common scale. The normalization method used is the min–max normalization, which is a linear normalization technique that adjusts the intensity values in the CTA image based on the minimum and maximum intensity values. This technique is effective for standardizing the image data and reducing the effects of image acquisition and reconstruction artifacts.

### 2.3. Heart Segmentation (Region of Interest)

Extracting the region of interest (ROI) by segmenting the heart from CTA images has a significant impact on the segmentation of coronary arteries. The essential ROI extraction process involves accurately identifying and segmenting the heart region from surrounding tissues, a critical step in segmenting coronary arteries located within the heart amidst other tissues and structures.

Segmenting the heart region of interest (ROI) from CTA images enhances the computational efficiency of the coronary artery segmentation process. By focusing on the heart region, the deep learning models can reduce the amount of data that needs to be processed, leading to faster segmentation times and decreased computational resource requirements. This is particularly beneficial in the context of medical imaging, where large datasets and high-resolution images are common. Furthermore, ROI segmentation can also improve the accuracy of the segmentation process by reducing the foreground-background imbalance problem. By focusing on the heart region, the deep learning models can decrease the number of background pixels, which can enhance the accuracy of the segmentation process [20,21].

In this work, the proposed framework employs two cascaded Unet-based models. The first model is responsible to localize and segment the heart organ from the target CTA scan. The first Unet-based model has been designed to capture the complex structures and patterns in medical images, enabling accurate segmentation of the heart region from the CTA images. By accurately segmenting the heart region, the second model can focus on the segmentation of the coronary arteries within the heart, improving the accuracy and efficiency of the segmentation process.

### 2.4. Vesselness Enhancement

Enhancing coronary arteries in coronary CT angiography (CTA) images is crucial for accurate diagnosis and treatment planning in cardiovascular medicine. In deep learning systems, the enhancement of the intensity of the coronary arteries compared to the surrounding tissues improves their appearance and enables the deep learning methods to learn the clear features of them. The Frangi vesselness filter has emerged as a sophisticated technique to address the challenge of low contrast-to-noise ratio inherent in coronary CTA datasets.

Frangi vesselness filtering operates by leveraging the Hessian matrix to analyze the local curvature of structures within the image. By convolving the input image with 3D Gaussian filters at multiple scales, the filter detects structures of varying sizes, which is crucial for capturing the complex network of coronary arteries spanning from large vessels to minute branches. The filter then assesses the eigenvalues of the Hessian matrix at each voxel, assigning a vesselness score that indicates the likelihood of the voxel belonging to a tubular structure, such as a coronary artery [22]. Convolution with 3D Gaussian filters at various scales is performed on the images, and the eigenvalues of the Hessian matrix at each pixel or voxel are examined using a response function to characterize the local structures within the images. The discrimination function, known as vesselness, is evaluated for each scale σ, representing the vessel radius, following the principles outlined in Equation (1).

(1) $$v\left(\sigma \right)=\{\begin{array}{c} 0,\;\lambda_{2}\geq 0,\lambda_{3}\geq 0\\ \left[1-e^{\left(-R_{a}^{2}/2\alpha^{2}\right)}\right]e^{\left(-R_{b}^{2}/2\beta^{2}\right)}\left[1-e^{\left(-S^{2}/c\right)}\right],\;else \end{array}$$

where

$$R_{a}=\frac{\left|\lambda_{2}\right|}{\left|\lambda_{3}\right|}\;\mathrm{and}\;R_{b}=\frac{\left|\lambda_{1}\right|}{\sqrt{\left|\lambda_{2}\lambda_{3}\right|}}$$

and these are the parameters that differentiate tube-like structures from plate-like and blob-like structures, where *|λ1| ≤ |λ2| ≤ |λ3|* are the eigenvalues of the Hessian matrix in 3D. The Frobenius norm of the Hessian matrix, *S*, addresses the difference between vessels and the background. The constants *α*, *β*, and *c* tune the sensitivity of *R_α_, R_b_*, and *S*, respectively, in the vesselness Equation (1). In this paper, α and β are set to 0.5, while the value of c depends on the grayscale range of the image, with the optimal value being half the maximum value of the Hessian norm [23].

**Figure 2:**
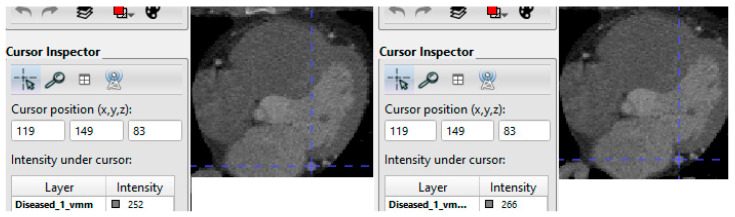
Coronary arteries vessels’ appearance: original (**left**) vs. enhanced (**right**).

Fine-tuning the parameters of the Frangi vesselness filter is essential for optimal coronary artery intensity enhancement. Parameters such as the scale of the Gaussian filters and the sensitivity of the discrimination function are adjusted to maximize coronary artery contrast while minimizing noise and artifacts. The Hessian enhancement is applied to the target image at multiple scales on each voxel to calculate the maximum vesselness values. Enhancing small vessels’ contrast is achieved by the Hessian filter at scale values of 1–3, while vessels with large radii benefit from enhancement at scale values of 4–8 [22]. This optimization process ensures that the enhanced images provide clear and detailed visualization of coronary arteries, facilitating accurate diagnosis and treatment decisions. Figure 2 shows the effect of applying vesselness and the intensity enhancement within the coronary arteries lumens, which is captured using the ITK-SNAP visualization software. In this study, the vesselness was applied with the scale values range (2–8) to make sure the coronary arteries are improved at different radii values [24].

The clinical implications of enhanced coronary artery visualization are profound. Accurate assessment of coronary artery stenosis, detection of coronary artery anomalies, and planning of interventional procedures such as percutaneous coronary intervention (PCI) or coronary artery bypass grafting (CABG) rely heavily on the clarity and accuracy of coronary CTA images. Moreover, in the building of our automatic computer-aided systems, the improved visualization of coronary arteries enables research into the pathophysiology of coronary artery disease (CAD) and the development of novel diagnostic and therapeutic approaches, like deep learning-based computer aided segmentation systems.

### 2.5. Network Architecture

**Figure 3:**
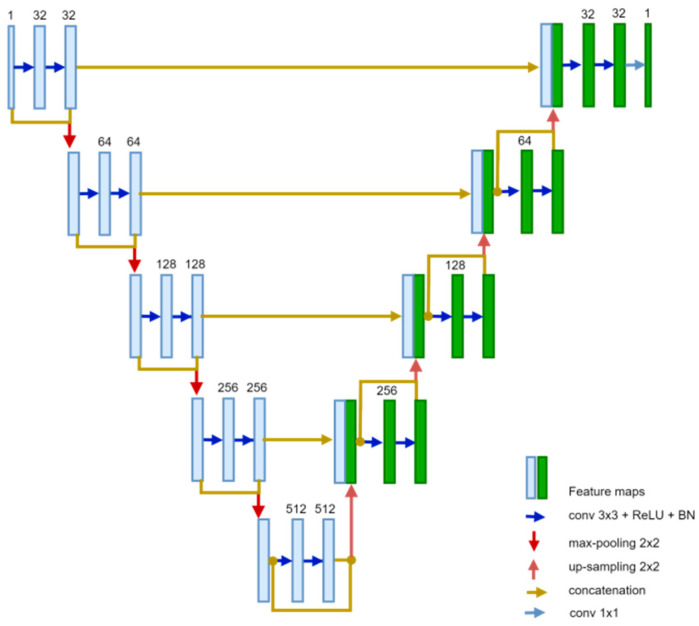
The proposed U-net-based model.

The framework consists of two cascaded U-net-based models, the first to localize and segment the heart organ from the target CTA. While the second model use the vesselness-enhanced heart region of interest as input to segment the coronary arteries. For this aim, in this study, we employed a deep Fully Convolutional Network (FCN) to conduct segmentation of coronary arteries. The FCN architecture is constructed based on the U-net design, featuring five hierarchical levels [25], illustrated in Figure 3. The U-net architecture comprises an encoding path and a decoding path. Within the encoding path, each level undergoes three consecutive operations: convolution, Rectified Linear Unit (ReLU) activation, and batch normalization. These operations are iterated twice within each level block, followed by max-pooling to down-sample the feature maps. Convolutional operations employ 3 × 3 kernels, while max pooling utilizes 2 × 2 kernels, leading to a reduction in feature map resolution at each level.

In the decoding phase, the initial input dimensions are reconstructed symmetrically. This involves applying the same sequence of operations as in the encoding phase (convolution, ReLU, batch normalization), replacing max pooling with up-sampling. Furthermore, the feature maps from matching levels in the encoding phase are merged with the input of each decoding level. The ultimate step in the decoding phase involves a 1 × 1 convolutional layer with a sigmoid activation function, aiding in classifying the feature map and producing the ultimate binary prediction map for coronary artery segmentation.

The expansion of network depth within Fully Convolutional Networks (FCNs) is unavoidable, yet it gives rise to the challenge of the vanishing gradient problem. This occurs when numerous layers are stacked together, causing the gradient information to diminish and impede effective training, thereby diminishing performance. Various deep network architectures have been proposed to address this issue, with DenseNet and ResNet emerging as pivotal advancements in terms of performance.

**Figure 4:**
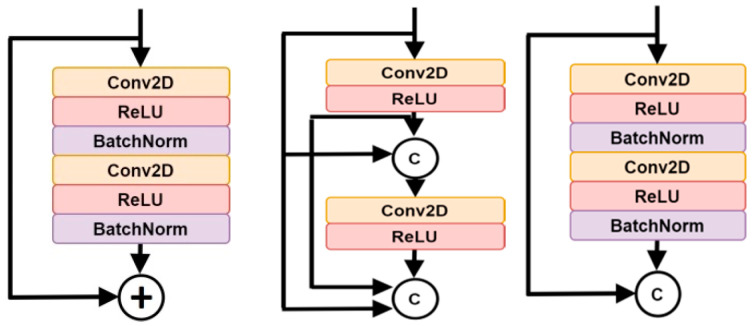
Residual block (**left**), dense block (**middle**), and ResDense Block (**right**).

In DenseNet, each layer establishes connections with all subsequent layers, enabling the fusion of feature maps from earlier layers with different filter sizes. This results in a significant increase in the model’s width as channels are combined after each convolution step [26]. On the other hand, ResNet takes a different approach by using an addition operation to combine the previous input identity with the output feature map. In a ResNet block, a shortcut (or skip connection) from the block’s input (identity) bypasses the stacked layers and merges with the block’s output feature [27]. Figure 4 illustrates the contrast in connections for Residual block (left) and Dense block (middle).

DenseNet optimizes the flow of information between layers by concatenating features rather than summing them, as seen in ResNet. As a result, DenseNet is considered a memory-intensive network, requiring the storage of entire layer outputs during back-propagation, leading to higher memory usage and slower execution times. On the other hand, ResNet prioritizes the direct addition of tensors, although there have been discussions about its potential impact on gradient flow throughout the network, given that it sums up feature values.

The preference for concatenation over summation arises from its ability to maintain feature maps, while summation can potentially distort feature maps in convolution operations and skip connections. A notable advancement in the network architecture is the introduction of the ResDense block, as shown in Figure 4 (middle). Within the ResDense block, dense connections (concatenation) link residual blocks instead of convolution layers, enhancing feature map flow and memory utilization. This strategic design optimizes feature value refinement through residual blocks and intermittently retains refined feature values via dense connections among residual blocks. The proposed FCN in this research integrates ResDense blocks in both the encoder and decoder paths. Each level in the contracting and expanding paths of the network incorporates ResDense blocks, as depicted in Figure 4 (right). Consequently, the feature map depth is doubled, and the resulting feature map is concatenated with the block input at the end of each level in the encoding path.

### 2.6. Model Implementation, Training and Loss Function

The proposed Unet-based network was trained on labeled 2D slices, utilizing patch dimensions of 256 × 256 extracted from the segmented heart region of interest. Patch-wise normalization was applied to all CTA slices, utilizing zero mean and unit variance normalization. The architecture of the U-net model was developed using Keras with the TensorFlow backend. The first model aims to localize and segment the heart organ from the target CTA automatically. The 2D slices used for training represent the whole area of the axial slices in the CTA scan. On the other hand, the second model uses the slices generated from the region of interest defined by the segmented heart in the previous stage.

**Figure 5:**
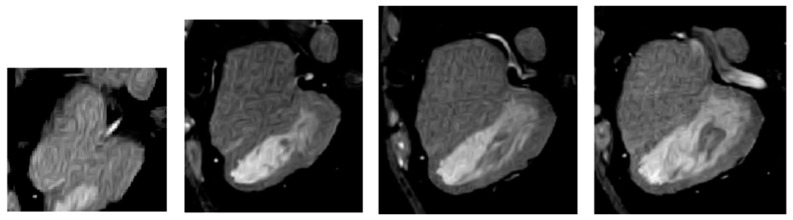
Training patches (heart region).

To address the imbalanced class distribution of vessel areas, various strategies can be implemented to enhance the trained network’s performance. The training process incorporates a combined binary cross-entropy (BCE) and soft dice loss (DSC) with equal weights as the loss function to evaluate the agreement between ground-truth patches and regions identified as vessels by the network within the heart ROI. The network was specifically trained within the heart ROI to differentiate features that distinguish vessels from the surrounding heart tissue. Training patches were obtained by isolating the heart region from the CTA slices, as illustrated in Figure 5, ensuring that each training patch had a corresponding mask; patches without annotation masks were excluded from the training dataset.

### 2.7. Performance Measures

In the field of medical image segmentation, evaluating the performance of deep learning-based models is crucial for assessing their accuracy and reliability. Three of the most widely used metrics in this domain are the Dice Similarity Coefficient (Dice), Precision, and Recall.

First, the Dice Similarity Coefficient (Dice) is a metric that measures the overlap between the predicted segmentation and the ground truth segmentation. It is calculated according to the formula in Equation (2):

(2) $$DSC=\frac{2\left|A\cap^{\;}B\right|}{\left|A\right|+\left|B\right|}$$

where A and B represent the number of pixels in the predicted and ground truth segmentations, respectively, and |A ∩ B| represents the number of pixels that is common between the two segmentations. The Dice score ranges from 0 to 1, with 1 indicating perfect overlap between the predicted and ground truth segmentations.

Second, the Jaccard index, also known as the Jaccard similarity coefficient or Intersection over Union (IoU), is a metric used to evaluate the similarity between the prediction and ground truth. The Jaccard index measures the similarity between two segmentation masks by comparing the size of their intersection with the size of their union, as explained in Equation (3).

(3) $$JI=\frac{\left|A\cap^{\;}B\right|}{\left|A\right|\cup^{\;}\left|B\right|}$$

Precision and Recall are two complementary metrics that provide insights into the model’s performance. Precision measures the proportion of true positive predictions (TP) among all positive predictions, while Recall measures the proportion of true positive predictions among all actual positive instances. Precision is calculated as explained in Equation (4), while Recall is calculated using Equation (5).

(4) $$Precision=\frac{TP}{TP+FP}\;$$

(5) $$Recall=\frac{TP}{TP+FN}$$

True Positives (TP) are the pixels that are correctly identified as belonging to the target structure, False Positives (FP) are the pixels that are incorrectly identified as belonging to the target structure, and False Negatives (FN) are the pixels that are incorrectly identified as not belonging to the target structure. Precision and Recall are often used together to provide a more comprehensive evaluation of the model’s performance. A high Precision indicates that the model is making accurate predictions, while a high Recall indicates that the model is identifying a large proportion of the target structure.

In our proposed framework, the Dice, Precision, and Recall metrics are particularly important because they provide insights into the proposed model’s ability to accurately identify and delineate the coronary arteries from the target CTA. This information is crucial for clinical decision making and patient care. As the aim of this work is the segmentation of coronary arteries from CTA images, a high Dice score would indicate that the model is accurately identifying the location and boundaries of the coronary arteries, which is essential for the diagnosis and treatment of cardiovascular diseases. Similarly, high Precision and Recall values would indicate that the model is making accurate predictions and identifying a large proportion of the target structure, respectively.

## 3. Results and Discussion

This section presents the quantitative (numerical) and qualitative results for the main framework steps: heart segmentation and coronary arteries segmentation.

### 3.1. Experimental Setup

The performance of the deep learning models for heart and coronary artery segmentation from CTA images was evaluated using a comprehensive experimental setup. The CTA dataset was split into training, validation, and test sets, ensuring no overlap between them. An 80/20 split was used, with 80% of the data being used for training and 20% for validation. This allowed us to carry out model selection and hyperparameter tuning based on validation performance.

The deep learning models were implemented using the Keras framework. The base architecture was a 2D U-Net with four encoding and decoding blocks. Model training and parameter updates were conducted using the Adam optimizer, initializing with a learning rate of 0.0001. Throughout the training process, various monitoring hyperparameters were employed. If there was no improvement in the validation Dice similarity coefficient (DSC) over three consecutive epochs, the learning rate was decreased by a factor of 0.5. Additionally, training stopped if the validation DSC did not improve over five consecutive epochs. The networks were trained with a batch size of 16 and a maximum of 500 epochs. Gradient clipping with a maximum norm of 1.0 was used to stabilize training.

The final layer of the network employed a pixel-wise sigmoid activation function, followed by thresholding at 0.5 to obtain binary segmentation masks. Separate models were trained for heart and coronary artery segmentation, with the coronary artery model using a smaller input patch that represented the region of interest in the heart organ to accommodate the smaller structures.

To evaluate the models, the Dice similarity coefficient (DSC), Jaccard Index (JI), Sensitivity, and Precision were calculated between the predicted segmentations and ground truth annotations. These metrics were computed for the heart and coronary arteries separately. By following this rigorous experimental setup, including dataset splits, data preprocessing, model architecture, training hyperparameters, and evaluation metrics, the performance of the deep learning models in heart and coronary artery segmentation from CTA images was thoroughly assessed. The results presented in the next sections provide insights into the potential of these techniques for clinical applications.

### 3.2. Heart Segmentation

As is highlighted in the previous section, the segmentation of the heart organ from Computed Tomography Angiography (CTA) images is a crucial step in the proposed framework, as it provides vital information by giving the region of interest (ROI) for the diagnosis and segmentation of coronary arteries. In this context, the use of ResUNet, combined with effective preprocessing Coherence Enhancing Diffusion (CED) filtering, showed a significant improvement in the accuracy and reliability of the segmentation process.

The proposed method is evaluated quantitatively by measuring the values of the performance measures, the results have been exceptionally impressive. The Dice similarity coefficient (DSC) achieved a remarkable value of 0.98, indicating an extremely high overlap between the predicted heart segmentation and the ground truth. Additionally, the Recall and Precision metrics were equally outstanding, with values of 0.99 and 0.99, respectively.

**Figure 6:**
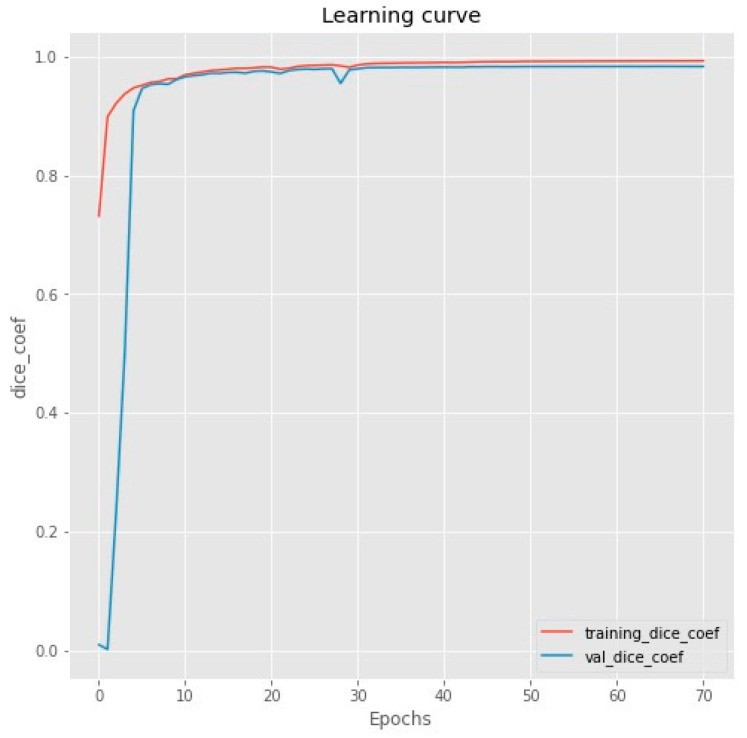
ResUnet model training curve for heart segmentation step.

To understand the training process and the model’s performance, it is essential to analyze the training curve. The training curve for the heart segmentation process showed a steady increase in the DSC metric as shown in Figure 6, reaching a plateau at around the 50th epoch. This indicates that the model was able to learn the complex features of the heart region effectively and converge to a stable and high-performing solution.

**Figure 7:**
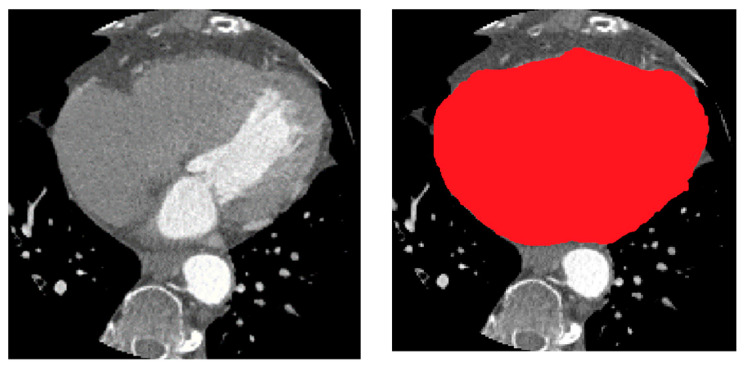
Example of heart segmentation from target CTA.

The high DSC score of 0.98 suggests that the ResUNet model was able to accurately identify and delineate the heart region within the CTA images. This is a significant achievement as the heart is a complex structure surrounded by other organs and tissues, making its segmentation a challenging task. The high DSC score demonstrates the model’s ability to capture the intricate details and boundaries of the heart, ensuring that the segmentation closely matches the ground truth. An example of the segmented heart (red color) in a CTA image is shown in Figure 7.

For other measures values, the Recall metric of 0.99 indicates that the model was able to identify almost all of the heart region, with only a negligible proportion of the heart organ being missed. This is a crucial aspect, as it ensures that the model is not overlooking important anatomical features that could be crucial for the next stage of segmenting the coronary arteries. The high Recall score suggests that the model is highly sensitive in detecting the heart region, which is particularly important in the next stage of segmenting the coronary arteries where missing critical information could have negative consequences. Furthermore, the Precision metric of 0.99 indicates that the model’s predictions were highly accurate, with a low rate of false positive detections. This means that the model was able to distinguish the heart region from the surrounding tissues and structures with a high degree of certainty.

### 3.3. Coronary Arteries Segmentation

The second part of the framework focus on segmenting the coronary arteries from the heart region of interest segmented in the previous section. The use of the proposed framework with and without a vesselness step is studied ad highlighted, which can significantly impact the accuracy and efficiency of the segmentation process.

For this purpose, many experiments have been carries to carry a comparative study between model with and without a vesselness step. The results have provided valuable insights into the performance of these models, as its outlined in Table 1. First, for the model with the vesselness step, the Dice Similarity Coefficient (DSC) achieved a value of 0.867, with Recall and Precision values of 0.881 and 0.892, respectively. On the other hand, ResUNet without the vesselness step yielded a DSC of 0.852, with Recall and Precision values of 0.858 and 0.871, respectively.

**Figure 8:**
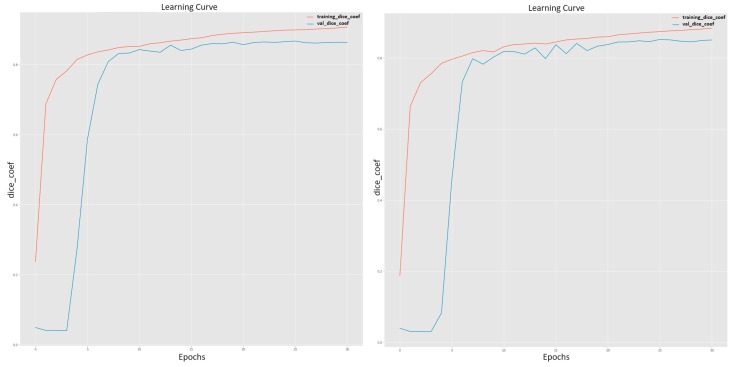
Proposed model training curves for coronary arteries segmentation: without vesselness (**top**); with vesselness (**bottom**).

Analyzing the training curves for both scenarios can provide further understanding of the proposed framework performance as shown in Figure 8. The training curve for the model with the vesselness experiment showed a steady increase in the DSC metric during training, reaching a plateau at around the 30th epoch. This indicates that the model was able to learn the features of the coronary arteries effectively and converge to a stable and high-performing solution. In contrast, the training curve for the model without the vesselness step exhibited a similar trend, albeit with slightly lower performance metrics, indicating that the model was still able to learn the features of the coronary arteries but with a slightly lower accuracy.

The higher values of DSC, Recall, and Precision achieved by the framework with the vesselness step suggest that incorporating vesselness information into the segmentation process succeeded to improve the model’s ability to accurately identify and delineate coronary arteries in CTA images. The vesselness enhancement step highlighted the vessel structures and enhanced their visibility, aiding the model in distinguishing the coronary arteries from surrounding tissues more effectively. On the other hand, without the vesselness, is the model demonstrated a good performance, but the slightly lower DSC, Recall, and Precision values indicate that the model may have faced challenges in accurately capturing the intricate details and boundaries of the coronary arteries. Without the vesselness enhancement, the model may have had to rely solely on image features, potentially leading to a slightly lower performance compared to the vesselness-enhanced model. In general, we can say that the results shown in Table 1 and the curves in Figure 8 highlight the impact of incorporating vesselness information into the segmentation process for segmenting coronary arteries from CTA images. The higher metric values achieved with the vesselness step demonstrated the importance of leveraging vessel enhancement techniques to improve the accuracy and efficiency of coronary artery segmentation.

**Figure 9:**
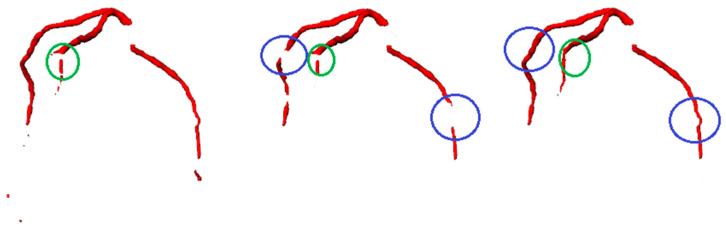
Coronary arteries masks: ground truth (**left**) without vesselness (**middle**) and with vesselness (**right**): the blue circles show the help of vesselness to improve the segmentation process. The green circle highlights the improving in the segmentation even better than the ground truth.

To qualitatively compare the results of segmenting coronary arteries in the target CTA images between the models with and without the vesselness step, a visual inspection of the CTA example is shown in Figure 9. As shown in the figure, the blue circles highlight how the vesselness enhancement helped the model to better identify and delineate the vessel structures, leading to a more accurate segmentation of the coronary arteries. It is clear that the vesselness step enhanced the visibility and contrast of the vessel structures, making it easier for the deep learning model to distinguish the coronary arteries from the surrounding tissues and background. When comparing the segmentation results to the ground truth mask, the predicted mask generated by the model with the vesselness step exhibits a clearer and more detailed delineation of the coronary arteries, including the main vessels and smaller branches. The vessel lumens are likely to be more accurately captured, with better preservation of the intricate vascular network.

In contrast, the predicted segmentation mask from the model without the vesselness step shows slightly lower accuracy in capturing the fine details of the coronary arteries as shown by the blue circles, potentially missing or blurring some of the smaller vessel branches. The vessel lumens may not be as clearly defined, and the overall vascular structure may appear to be less detailed compared to the ground truth. The green circles highlight that the improved performance of the vesselness-enhanced ResUNet model proves the importance of incorporating vesselness step as it succeeded in capturing the disconnections of the vessels’ branches even in the ground truth mask, by enhancing the visibility and contrast of the vessel structures, which helped the model to better identify and delineate the complex coronary artery network, leading to more accurate and reliable segmentation results.

### 3.4. Previous Study

In the field of coronary artery segmentation from CTA images, researchers have explored various deep learning architectures and techniques to address the challenges and improve the accuracy of the segmentation process. A study by Song et al. proposed an efficient feature fusion and rectification 3D-UNet for automatic coronary artery segmentation in CTA images. The method leverages a 3D-UNet architecture with a feature fusion module and a rectification module to enhance the segmentation accuracy. The authors reported a Dice similarity coefficient (DSC) of 0.8795, highlighting the effectiveness of their approach [28]. Similarly, Tian et al. developed an automatic coronary artery segmentation algorithm based on deep learning and digital image processing. Their method combines a convolutional neural network (CNN) with traditional image processing techniques, such as adaptive thresholding and morphological operations, to segment the coronary arteries. The authors achieved a DSC of 0.8723, demonstrating the potential of hybrid approaches for this task [29].

Addressing the challenge of unstable image quality in CTA images, Wang et al. proposed a two-stage U-Net-based method for coronary artery segmentation. The first stage focuses on extracting the coronary artery region, while the second stage refines the segmentation using a more detailed U-Net model. This approach achieved a DSC of 0.8954, highlighting the benefits of a multi-stage segmentation strategy [30]. Furthermore, researchers have explored the integration of attention mechanisms to enhance the performance of deep learning models for coronary artery segmentation. Vaswani et al. introduced the Transformer architecture, which utilizes self-attention to capture long-range dependencies in data. This concept has been adapted for medical image segmentation, as demonstrated by Lei et al., who developed an automated coronary artery segmentation method using attention-based deep learning networks [15].

Another study by Song et al. proposed an efficient feature fusion and rectification 3D-UNet for automatic coronary artery segmentation in CTA images. The method leverages a 3D-UNet architecture with a feature fusion module and a rectification module to enhance the segmentation accuracy. The authors reported a Dice similarity coefficient (DSC) of 0.8795, highlighting the effectiveness of their approach [28].

Furthermore, researchers have explored the integration of attention mechanisms to enhance the performance of deep learning models for coronary artery segmentation. Vaswani et al. introduced the Transformer architecture, which utilizes self-attention to capture long-range dependencies in data. This concept has been adapted for medical image segmentation, as demonstrated by Lei et al., who developed an automated coronary artery segmentation method using attention-based deep learning networks [31].

In addition to deep learning-based approaches, some studies have investigated the use of hybrid techniques that combine deep learning with traditional image processing methods. For example, Mihalef et al. proposed a method that integrates a level set function with deep learning for accurate coronary artery segmentation. This approach leverages the strengths of both techniques to address the challenges in CTA image analysis [32].

Gao et al. proposed an automatic approach to segment the three-dimensional coronary tree from computed tomography angiography (CTA) images. The method combines a learning-based approach with a graph-cut optimization to segment the vessels. The results showed that the method can accurately segment the major coronary arteries and their branches, providing a comprehensive analysis of the coronary tree [29].

Wolterink et al. developed a graph neural network approach for automatic extraction and labeling of the coronary artery tree from CTA images. The method uses a deep learning model to predict the presence of coronary arteries and their labels at each voxel. The predicted labels are then used to construct a graphical representation of the coronary tree. The study demonstrated the effectiveness of graph neural networks in accurately extracting and labeling the coronary arteries [16].

The related works highlight the significant progress made in the field of coronary artery segmentation from CTA images, with deep learning-based methods, such as Res-UNet and V-Net, demonstrating impressive performances. The integration of vesselness features, attention mechanisms, and hybrid approaches have further enhanced the accuracy and robustness of these segmentation techniques. Additionally, the exploration of graph-based methods for comprehensive coronary tree analysis provides a complementary perspective to the field.

### 3.5. Comparison with Different Segmentation Models

To evaluate the effectiveness of the proposed coronary arteries segmentation framework, it was compared with other cutting-edge methods that are commonly used for medical image segmentation. A detailed quantitative comparative analysis of the performance of the proposed framework against other models provides valuable insights into their performances in segmenting the coronary arteries from medical images based on their respective metrics values presented in Table 2.

The proposed framework with preprocessing vesselness and heart segmentation steps demonstrates a superior performance across multiple metrics compared to the other models. With a Dice similarity coefficient (DSC) of 0.867, it surpasses 3D-UNet (DSC = 0.83), VNet (DSC = 0.83), and UNetR (DSC = 0.82), indicating a higher level of overlap between the predicted and ground truth segmentations. This suggests that the combination of vesselness and heart segmentation preprocessing steps has significantly enhanced the model’s accuracy in delineating the heart region in medical images.

Furthermore, the proposed framework with vesselness and heart segmentation preprocessing steps exhibits a Recall of 0.881, outperforming VNet (Recall = 0.81) and UNetR (Recall = 0.78), indicating its ability to capture a larger proportion of true positive instances. Additionally, the precision of 0.892 for this model exceeds that of VNet (precision = 0.82), highlighting its accuracy in correctly identifying positive instances with fewer false positives. Compared to AttUNet with a DSC of 0.84, Recall of 0.83, and Precision of 0.85, the proposed framework demonstrates a competitive performance, showcasing its effectiveness in accurately segmenting the coronary arteries. In summary, the incorporation of vesselness and specific preprocessing steps has proven to be a valuable enhancement, significantly improving the model’s accuracy and reliability in segmenting the heart from medical images.

## 4. Conclusions

The proposed method, which includes vesselness enhancement, heart ROI extraction, and the ResUNet deep learning architecture, can effectively identify and delineate coronary arteries in CTA images. The vesselness enhancement step improves visibility of coronary arteries, enabling the intricate details to be captured better by the deep learning model. Clinically, this method can significantly enhance the accuracy of cardiovascular disease diagnosis and treatment, potentially leading to improved patient outcomes.

## Figures and Tables

**Table 1:** Segmentation measures for coronary arteries with and without vesselness.

Method	DSC		Recall		Precision	
	Testing	Training	Testing	Training	Testing	Training
No vesselness	0.852	0.884	0.858	0.873	0.871	0.880
With Vesselness	0.867	0.907	0.881	0.893	0.892	0.91

**Table 2:** Comparison of coronary arteries segmentation with various methods.

Method	DSC	JI	Recall	Precision
3D-UNet [33]	0.837	0.719	0.844	0.837
VNet [34]	0.837	0.720	0.810	0.872
AttUNet [35]	0.843	0.730	0.835	0.857
UNETR [36]	0.827	0.710	0.784	0.884
Proposed Method	**0.867**	0.765	**0.881**	**0.892**

## Data Availability

The data used in the study are publicly available in [ASOCA challenge] at [https://asoca.grand-challenge.org/], accessed on 21 July 2024 and [MM-WHS: Multi-Modality Whole Heart Segmentation] at [https://zmiclab.github.io/zxh/0/mmwhs/], accessed on 21 July 2024.
